# Non-Male Factor Only—ICSI Can Overcome Oocyte Factor in PCOS Patients

**DOI:** 10.3390/jcm14010244

**Published:** 2025-01-03

**Authors:** Yasmin Shibli Abu Raya, Nardin Aslih, Yuval Atzmon, Moamina Sharqawi, Maya Shavit, Asaf Bilgory, Einat Shalom-Paz

**Affiliations:** 1 Gynecological Research Laboratory, Department of Obstetrics and Gynecology, Hillel Yaffe Medical Center, Hadera 3820302, Israel; nardin_aslih@yahoo.com (N.A.); atzmony@gmail.com (Y.A.); moaminash@gmail.com (M.S.); maya.genel@gmail.com (M.S.); asaf_bil@hotmail.com (A.B.); 2 Ruth and Bruce Rappaport School of Medicine, The Technion Institute of Technology, Haifa 3200003, Israel

**Keywords:** IVF, ICSI, KIDScores, PCOS, sibling oocytes, morphokinetics

## Abstract

**Background:** In this research, we retrospectively studied the influence of the IVF vs. the ICSI technique on embryo morphokinetics by means of a time-lapse incubator in fresh cycles. **Methods:** A total of 2645 treatment cycles resulting in ovum pick-up of 11,471 fertilized oocytes were included in the research from 2018 to 2022. The embryos were grouped according to IVF or ICSI. Embryonic development was monitored using a time-lapse incubator, and they were transferred on day 3 or 5. **Results:** The embryos in the ICSI group developed faster and had less fragmentation. However, fewer 2PNs were achieved and more embryos were discarded compared to IVF. When sibling oocytes treated with either IVF or ICSI were analyzed, we found that ICSI resulted in quicker development and higher KIDScores. Discussion: The anovulation and PCOS subgroups were the primary contributors to the high KIDScores in sibling oocytes, indicating that ICSI might have beneficial effects on oocyte factors, similar to the positive results it provides when male factors are involved. **Conclusions:** Women with PCOS undergoing IVF had better results when ICSI was used compared to spontaneous IVF. This study reveals that ICSI is superior to IVF in female factor infertility.

## 1. Introduction

Intracytoplasmic sperm injection (ICSI) was first used to overcome male factor infertility, while in vitro fertilization (IVF) was reserved for non-male factor indications, including anovulation, endometriosis, unexplained, and tubal factor [1]. Age was not found to be an indication for ICSI [2]. Recently, the use of ICSI for non-male factor indications has increased substantially. However, a concomitant increase in live birth rates has not been observed [3].

With the goal of obtaining the best pregnancy rate per transfer, extensive efforts have been made to define the best-quality embryos [4]. Morphokinetics, using a time-lapse incubator system, is more informative in comparison to traditional methods, in which embryo quality is assessed under a microscope [5]. In a time-lapse incubator, the embryo’s morphology is continuously monitored, and images are taken automatically at consistent intervals, without removing the embryo from the incubator. This provides the embryo with a stable, culture environment with the fewest variations, including temperature and gas concentration [6]. Moreover, continuous monitoring of the embryo reveals developmental patterns that could help identify top-quality embryos with high growth potential [7].

Initial evidence indicates some differences in the morphokinetics of a developing embryo based on the fertilization method. It was found that the 2-cell stage of IVF embryos was longer than that of ICSI embryos [8]. In addition, the 3-cell stage of IVF embryos was 1.3 h longer than that of ICSI embryos [8].

ICSI is thought to prevent about 30% of fertilization failures [9]. These data are consistent with those from the European Society of Human Reproduction and Embryology, showing that ICSI was performed in 71% of cycles and resulted in a significant increase in fertilization and pregnancy rates [10]. A recent study comparing ICSI with IVF in women of advanced age, with diminished ovarian reserve and low number of oocytes, did not lead to an increase in clinical pregnancy and cumulative live birth rates despite a higher normal fertilization rate [11].

The decision on whether to use IVF vs. ICSI is based mainly on sperm parameters. However, in the absence of male factor infertility, data on whether ICSI is more beneficial than conventional insemination conflict [3]. Some studies support wider use of ICSI [12], while others found that conventional insemination has better results [13,14].

Information on the impact of conventional IVF vs. ICSI on embryo morphokinetics is scarce. Therefore, the present study used a time-lapse incubator and assisted reproductive technique outcomes to evaluate whether the fertilization method affects embryonic development. The primary outcome was the KIDScore, and the secondary outcomes were developmental points during embryo morphokinetics and the final utilization of each embryo (transfer, freeze, or discard).

## 2. Methods

### 2.1. Study Design

This study involved retrospective analysis of prospectively collected data of all fresh assisted reproductive technique cycles, including data from the time-lapse system at the IVF Unit in the Hillel Yaffe Medical Center from 2018 to 2022. The Institutional Review Board of the Hillel Yaffe Medical Center approved the study (number 26-20HYMC) on 27 April 2020. As this was a retrospective study, informed consent was waived.

Inclusion criteria were all patients undergoing IVF or IVF-ICSI with images of morphological and morphokinetic patterns of embryo development in fresh cycles obtained using time-lapse technology. Fertility preservation cycles were excluded.

### 2.2. Participants

**Figure 1:**
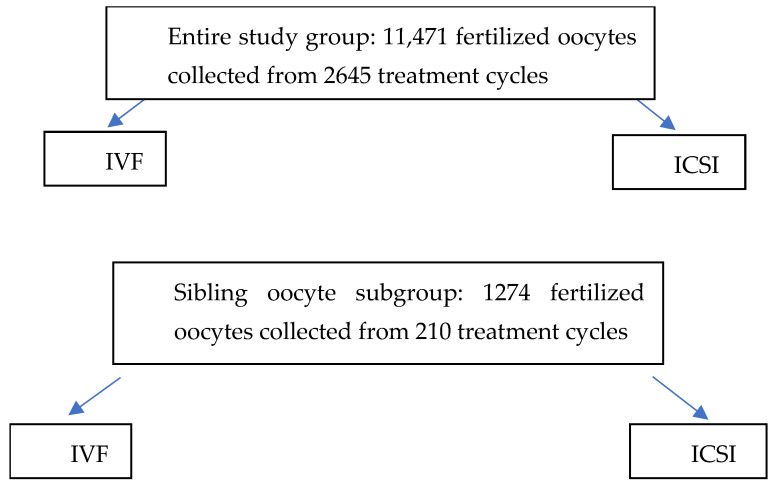
Graphical representation of the entire study group and the sibling oocyte subgroup.

This study evaluated all embryos that were incubated in a time-lapse system. Embryos were collected from 1413 patients who underwent 2645 treatment cycles (Figure 1). In the entire study group, oocytes from each patient were fertilized using IVF only, ICSI only, or half with ICSI and half with IVF. The developmental events of the embryos were investigated. To determine the best fertilization methods during the first IVF treatment for non-male factor, in cases where we retrieved at least two mature oocytes (M2) to fertilize, our practice is to divide the oocytes into 2 sibling groups: IVF-insemination and ICSI. We analyzed this subgroup of patients based on the etiology of infertility. When there is one oocyte, we usually prefer ICSI.

A subgroup of sibling oocytes collected from 210 treatment cycles (Figure 1) that were inseminated using ICSI or IVF in the same cycle were analyzed separately.

Clinicians decided on the protocol type and medication doses based on patient characteristics in each fresh cycle. Results are presented in Table 1 and Table S1. Doses were adjusted depending on the ovarian response, as assessed by ultrasound scans and estradiol blood levels. After ovum pick-up, oocytes were fertilized either by injecting a single sperm directly into the oocyte (ICSI) or by conventional IVF insemination, in which the oocyte is surrounded by sperm for fertilization in the laboratory (IVF). On the day of the transfer, day 3 embryos or blastocysts were transplanted with the assistance of abdominal ultrasound.

### 2.3. Embryonic Evaluation Using Embryoscope Images

Embryos were incubated in the Embryoscope for 120 h, which allows embryologists to monitor embryo cell division while the embryos are still in the incubator. Images of the embryos were received using EmbryoViewer software Ver.7.9.4 (Unisense FertiliTech, Aarhus, Denmark). The time points included in the study were defined as tPB2 (time to second polar body extraction), tPNa (time to pronucleus appearance), tPNf (time of pronucleus fading), cleavage time (time of 2PN cell division to two completely separate cells), and t2, t3, t4, t5, t8 represented the time points of a blastomere embryo reaching the stages of 2, 3, 4, 5, and 8 cells, respectively. Other parameters analyzed were tSC, time to start compaction; tM, time to morula; tSB, time to starting blastulation; tB, time to blastocyst; tEB, time to expanded blastocyst; and tHB, time to hatched blastocyst.

Embryo quality scoring was defined based on the KIDScore, according to the time-lapse model (Vitrolife^®^, Ottawa, ON, Canada) and a set of criteria for embryo morphology staging, as defined in the Istanbul consensus [15]. Accordingly, the optimal oocyte morphology was that of a spherical structure enclosed by a uniform zona pellucida, with a uniform translucent cytoplasm, free of inclusions, and a size-appropriate polar body. For embryos, each observation was recorded according to cell number/staging and grading, each of which was reported separately [15]. The KIDScore is a model based on the morphology and morphokinetic parameters of embryos.

Top-quality embryos on day 3 included KIDScores of 5 or 4. After embryo transfer, high-quality embryos were either vitrified for future use in frozen embryo transfer cycles or incubated until day 5. The usual protocol in our clinic is day 3 embryo transfers, based on the pregnancy rate with cleavage stage embryos. Low-quality embryos on day 3 are incubated until day 5. The decision to discontinue the incubation and discard the embryos is based on their quality.

A pregnancy blood test was performed 12 days after embryo transfer, and if the result was positive, an ultrasound was performed 2 weeks later. If a gestational sac with a fetal heartbeat was detected by ultrasound, clinical pregnancy was confirmed.

Data that were considered in the analysis included demographic parameters (age, BMI, parity, and cause of infertility), cycle parameters (basal FSH and LH, treatment protocol, endometrial thickness, number of transferred embryos, and embryo quality), and treatment outcomes (chemical pregnancy or clinical pregnancy, and the spontaneous abortion rate). The outcome based on the fertilization method included the following measures: embryo morphokinetics and embryo quality based on KIDScore (as defined previously) and pregnancy results, including abortion. Demographic data, treatment information and results, and pregnancy outcomes were recorded and followed until delivery.

The data were analyzed in two steps. Initially, the entire group of M2 oocytes was divided according to the fertilization method of IVF vs. ICSI, and the resulting embryos were compared. Thereafter, the analysis focused on the group of sibling oocytes that were fertilized using IVF or ICSI. This group was analyzed according to the cause of infertility to determine which fertilization method had better results in each subgroup.

### 2.4. Statistical Analysis

Data were analyzed using SPSS, version 25 (IBM Corp., Chicago, IL, USA). The distribution of the data was determined using the Shapiro–Wilk test. Comparisons were performed using the Student’s *t*-test or the Mann–Whitney U test, each as appropriate. Proportions were compared using the Chi-square test or the Fisher exact test. *p*-values ≤ 0.05 were considered significant.

## 3. Results

A total of 11,471 fertilized oocytes collected from 2645 patients were evaluated using the EmbryoScope™ (Unisense FertiliTech, Aarhus, Denmark). Of these, 2085 (18.2%) oocytes were fertilized by oocyte insemination (conventional IVF group), and 9386 (81.8%) were fertilized by intracytoplasmic sperm injection (ICSI group).

### 3.1. Results of the Entire Group Divided According to IVF or ICSI

Patients in the IVF and ICSI groups were comparable in BMI, age, and treatment protocols (Table 1). The choice of fertilization method differed significantly according to the cause of infertility: ICSI was used more frequently for male factor cases compared to IVF (37.5% vs. 22.7%, respectively, *p* = 0.002). IVF was used more frequently than ICSI for cases of anovulation (14.5% vs. 8.1%, respectively, *p* = 0.031) and unexplained infertility (47.3% vs. 37.6%, respectively, *p* = 0.047).

In the IVF group compared to the ICSI group, there were more cases of normal fertilization, defined as embryos with production of 2PN (98% vs. 89.9%, *p* = 0.001), more embryos were either eligible to freeze or to transfer (92% vs. 83.5%, *p* = 0.001) and fewer were discarded (8% vs. 16.5%, *p* = 0.00). However, more cases of fragmentation were reported during the cleavage stage in the IVF group compared to ICSI (89.6% vs. 86.2%, respectively, *p* < 0.001). The KIDScores were comparable between the groups (Table 2).

The ICSI group demonstrated significantly faster development from fertilization to the morula stage based on the morphokinetics at the time points evaluated. From morula to blastocyst, the developmental rates were equivalent (Table 3).

### 3.2. Results of the Subgroup of Patients with Sibling Oocytes Treated with IVF or ICSI

A total of 1274 sibling oocytes were collected from 210 women. Demographic parameters of women were age 35.9 years, BMI 26.4 (kg/m^2^), FSH 7.9 (IU/L), LH 6/07 (IU/L), E2 53.1 (pg/mL), and P (0.26 (ng/mL). Infertility causes were unexplained (45.9%), PCOS (8.2%), endometriosis (5.7%), mild male factor (7.2%), tubal factor (15.3%), and combined (17.7%). Treatment protocols were antagonist (81.7%), long agonist (6.7%), short agonist (10.6%), and other (1%). A mean of 9.8 oocytes was collected per patient, and the pregnancy rate was 59%. As women contributed oocytes to both techniques, these parameters were similar in both groups. Of these oocytes, 756 were fertilized using ICSI and 518 using oocyte insemination (Table S1).

In the sibling oocyte subgroup analysis, we found that the embryonal development rate was faster in the ICSI group at all time points, starting from 2PN until the tSB stage (Table 4).

The KIDScore was higher in the ICSI group compared to the IVF group (3.68 vs. 3.35, respectively, *p* < 0.001; Table 5), and more frozen embryos were achieved in the ICSI group compared to the IVF group (34.2% vs. 28.2%, respectively, *p* = 0.02). It is clear that more embryos were discarded in the IVF group in the sibling subgroup when no sperm factor was involved (Table 5).

In a sub-analysis of the KIDScores based on the cause of infertility in the sibling oocyte group, the anovulating PCOS subgroups had significantly higher KIDScores with ICSI compared to IVF (4.02 vs. 3.37, *p* < 0.001). However, the KIDScores in all other subgroups (endometriosis, mild male factor (defined as concentration between 10 million/mL and 14 million/mL), tubal factor, and unexplained infertility) were similar (Table 6).

## 4. Discussion

This study evaluated the impact of the oocyte fertilization method (IVF or ICSI) on embryo morphokinetics. The cohort was analyzed based on the fertilization method, and subgroups were analyzed according to sibling oocytes and the cause of infertility. Interestingly, more normal fertilizations defined as embryos with two pronuclei (2PN) and more embryos available for use, resulted from IVF insemination compared to ICSI; however, with comparable embryonic quality, assessed by the KIDScores in both groups. More cases of fragmentation were detected in embryos after IVF compared to ICSI in the entire group and the developmental rate was faster both in the entire group and the sibling oocyte group in the ICSI group.

On the other hand, in the sibling oocytes analysis, embryos produced using ICSI had higher KIDScores compared to IVF insemination. When investigating the effect of the cause of infertility on embryo development using each mode of fertilization, we found that embryos from women in the anovulatory PCOS subgroup fertilized using ICSI had higher KIDScores compared to IVF.

ICSI was first introduced into IVF labs for severe male factor [16], and since then, its use has expanded widely. It is largely used for in vitro maturation cases where a reliable, timed insemination method is used to overcome the hardness of the zona pellucida [17]. In spontaneous fertilization, sperm induce cumulus cells to release specific cytokines and chemokines that bind to the sperm and enhance fertilization. Hence, after removing the cumulus and exposing the zona pellucida to confirm that the nucleus is mature, ICSI is needed to complete the fertilization process [18].

Previous studies found an association between congenital anomalies and assisted reproduction, with a higher risk of neonates conceived through ICSI being affected in comparison to IVF, especially for urogenital and musculoskeletal anomalies [19]. In fact, many centers use ICSI almost exclusively as a method of fertilization. This present study aimed to define indications for ICSI in cases of non-male infertility. Identifying the proper fertilization technique per oocyte according to the different maternal causes of infertility might enable us to offer personalized treatment. Maternal age did not affect the outcomes based on the fertilization method.

### 4.1. Normal Fertilization

Our findings of significantly more normal fertilization (2PN) and more usable embryos suitable for freezing or transfer in the IVF group compared to the ICSI group support those of other reports [20,21]. It was demonstrated that cumulus cells provide soluble factors that assure the selection of better male gametes. Exposing the sperm to the secretome of cumulus cells allows improved acrosome reaction, sperm capacitation, and mitochondrial activity [22]. Moreover, ICSI is more invasive than other spontaneous fertilization techniques, and there is a higher risk of damage to the oocyte from lysis or shrinkage [23]. Furthermore, the impact of the ICSI manipulation, which may be conducted too early in the oocyte developmental stage, and the fact it forces early penetration of the sperm into the cytoplasm of immature oocytes remains unclear [24].

Significantly more fragmentation was detected in embryos after IVF compared to ICSI. Several studies suggested that embryo quality might be affected by apoptotic cumulus granulosa cells, leading to fragmentation. Therefore, the surrounding apoptotic cells could be a cause of the increased fragmentation of embryos in IVF cycles [25]. The damage to the embryo by the apoptotic cells is induced by DNA fragments that are released into the follicular fluid. High levels of cell-free DNA in the follicular fluid and longer exposure of the oocyte to those apoptotic granulosa cells during IVF may damage oocyte quality and, consequently, reduce the embryo quality by higher fragmentation [26].

### 4.2. Developmental Rate and Embryo Quality

Oocytes fertilized by ICSI showed a faster cleavage rate both in the entire group and in the sibling oocyte group. As reported previously [27], injecting the sperm directly into the ooplasm during ICSI results in earlier pronuclear formation and first mitotic division. A possible explanation for earlier time points after ICSI compared to IVF is that ICSI embryos have an earlier starting point than IVF embryos. However, these differences disappear in the later stages of development [28]. To the best of our knowledge, no evidence-based explanation was reported for the disappearance of the differences in mean time points of embryonic development.

### 4.3. Fertilization Methods for Different Causes of Infertility

In a secondary analysis that categorized the sibling oocyte group based on the cause of infertility, PCOS was found to be the main subgroup that benefitted from using ICSI compared to IVF, resulting in embryos with significantly higher KIDScores. It is clear that more embryos were discarded after IVF in the sibling subgroup when no sperm factor was involved. We assume that ICSI overcomes different obstacles in embryo development that are referred to as oocyte factors, and IVF does not compensate for it such as in PCOS. Various explanations are proposed in the literature to understand the effect of PCOS on oocyte quality, such as insulin resistance [29], hyperandrogenism, increased oxidative stress and impaired mitochondrial energy metabolism [30], chronic inflammatory state, and lipid disturbances [31]. The association between peripheral insulin resistance and PCOS is well-known, resulting in impairment in target tissues. Abnormal insulin levels result in abnormal glucose uptake and may impair metabolic balance [32] and energetic pathways in the oocyte and the cleaved embryos and by that, lower oocyte quality and top-quality blastocyst formation rates [31]. Another effect of insulin in the follicle is increasing the local production of androgens through amplifying the influences of the LH on theca cells [33,34]. This causes premature development of follicles, which results in lower-quality oocytes [35]. We assume that the removal of the cumulus in ICSI should minimize oocyte exposure to the deleterious micro-environment caused by insulin resistance.

Another explanation could be related to insulin-like growth factor (IGF). This is a paracrine/autocrine polypeptide ligand secreted by the ovary that binds to the IGF receptor on the granulosa cells and regulates oocyte proliferation and maturation [36]. IGF1 is secreted during the LH surge and enables the maturation of oocytes depending on a mechanism involving an enzyme called a phosphoinositide-3-kinase combined with the v-akt murine thymoma viral oncogene homolog (PI3K/AKT). IGF-1R expression is higher in granulosa cells in PCOS, altering the level of miRNAs in their cumulus cells. These altered miRNAs could induce numerous genes that have different functions, regulating the signaling pathways like Wnt-, MAPK-, and PI3k/AKT, and regulate oocyte meiosis and maturation, proliferation of granulosa cells, and other aspects. Therefore, overstimulation of IGF1 by insulin may deteriorate oocyte quality and embryo development by the resumption of meiosis, resulting in post-maturity [37]. Early cumulus cell removal in ICSI would block intercommunication between cumulus cells and oocytes [38], which is essential for reducing the overactivation of IGF1R and blocking the negative effect of the hormonal imbalance surrounding the oocyte.

The expression of the zona pellucida 4 (ZP4) gene in polycystic ovaries is low compared to healthy controls, probably due to high levels of androgens that regulate the expression of zona pellucida genes [39]. The zona pellucida is a thin acellular interconnected filament surrounding oocytes and early embryos that is composed of four glycoproteins: ZP1, ZP2, ZP3, and ZP4. The zona pellucida mediates sperm–embryo interactions while the ZP4 subtype facilitates the acrosome reaction induced by N-glycosylation of the protein, along with phosphorylation of ion channels, followed by activation of a sustained extracellular calcium influx. In ICSI, the sperm is injected directly into the oocyte, penetrating the zona pellucida and bypassing the acrosome reaction [40]. Hence, ICSI can overcome hurdles resulting from the different zona pellucida of PCOS. Our results agree with the above, as we also demonstrated better treatment outcomes in PCOS patients.

## 5. Limitations

One of the limitations is the retrospective nature of the study, which may limit the impact of the results. However, the large cohort may overcome this. The lack of advanced pregnancy results such as live birth rate per fertilized oocyte may also have limited the interpretation. Another limitation is that fertility results might be influenced by factors other than the fertility technique, such as hormone levels, genetic factors, and environmental factors. However, these factors were eliminated in the analysis of the sibling oocytes subgroup.

### Strengths

This study included a large cohort of embryos and cycles. Moreover, it demonstrated the advantage of ICSI for patients with PCOS but not for other causes of infertility, with supporting biological rationale. An additional strength is the follow-up of each embryo until final utilization.

In conclusion, ICSI is suggested as the preferable mode of fertilization for the PCOS subgroup, resulting in embryos with higher KIDScores. This identification might enable us to improve treatment results by offering personalized care according to maternal causes. Removing the cumulus cells in ICSI might remove the negative hormonal disturbance surrounding the oocyte in PCOS. Our plan is to evaluate pregnancy outcomes, including the live birth rate, based on the fertilization method.

## Figures and Tables

**Table 1:** Demographic and treatment parameters of ICSI vs. IVF.

Oocyte Fertilization Method	ICSI (n = 9386)	Conventional IVF (n = 2085)	*p*-Value
Age (years)	35.5 ± 6.5	36.4 ± 6.5	0.13
BMI ≥ 18 (kg/m^2^)	26.5 ± 6.1	26.1 ± 6.8	0.082
Protocol			0.72
Antagonist	7080 (83%)	1488 (83%)	
Long agonist	965 (11%)	190 (11%)	
Short	515 (6.0%)	105 (6%)	
Cause of Infertility			
Anovulation	94 (8.1%)	16 (14.5%)	0.031
Male factor	436 (37.5%)	25 (22.7%)	0.002
Endometriosis	55 (4.7%)	3 (2.7%)	0.47
Unexplained	438 (37.6%)	52 (47.3%)	0.047
Tubal factor	141 (12.1%)	14 (12.7%)	0.85

**Table 2:** Embryonal development and KIDScore in the ICSI vs. IVF subgroups.

Fertilization Method	ICSI (n = 9387)	Conventional IVF (n = 2085)	*p*-Value
IRD (irregular division)	1506 (16%)	321 (15.4%)	0.48
Fragmentation	8087 (86.2%)	1869 (89.6%)	0.001
Mono-nucleation	7890 (84%)	1785 (85.6%)	0.08
Number of pronuclei			<0.0001
2 PN	8369 (89.9%)	2028 (98%)	
Others (0, 1, 3 and 4 PN)	668 (10.1%)	39 (2%)	
Number of embryos	(n = 5107)	(n = 909)	<0.001
Discarded	844 (16.5%)	74 (8%)	
Used (freeze or transfer)	4263 (83.5%)	835 (92%)	
KIDScore	3.3 ± 1.624	3.23 ± 1.571	0.08

**Table 3:** Mean time of morphokinetic points in ICSI vs. IVF of the entire group.

Time in Hours	ICSI	IVF	*p*-Value
tPNa	6.62	6.83	<0.001
tPNf	23.60	25.03	<0.001
t2	26.23	27.82	<0.001
t3	37.22	38.51	<0.001
t4	38.67	40.20	<0.001
t5	50.08	51.58	<0.001
t6	52.38	54.03	<0.001
t7	54.59	56.42	<0.001
t8	57.19	59.67	<0.001
t9	66.75	70.19	<0.001
tSC	83.74	86.78	<0.001
tM	91.64	92.97	<0.001
tSB	99.99	100.04	0.59
tB	108.60	108.86	0.91
tEB	115.00	115.25	0.41
tHB	116.18	122.8	0.41

Sperm concentration in the ICSI group was significantly lower than in the IVF group (20 MIL/mL vs. 45 MIL/mL, *p* < 0.001; Table S2).

**Table 4:** Mean time of morphokinetic points of sibling oocytes.

Time in Hours	ICSI	IVF	*p*-Value
tPN2	2.89	6.12	<0.001
tPNa	6.74	8.14	<0.001
tPNf	23.42	25.42	<0.001
t2	26.05	28.17	<0.001
t3	37.23	39.19	<0.001
t4	38.61	40.63	<0.001
t5	50.17	52.83	<0.001
t6	52.01	54.94	<0.001
t7	53.95	56.88	<0.001
t8	56.06	59.96	<0.001
t9	66.49	71.71	<0.001
tSC	82.36	88.30	<0.001
tM	91.52	94.88	<0.001
tSB	99.24	102.23	<0.001
tB	108.53	109.55	0.19
tEB	114.40	115.23	0.23
tHB	113.80	132.54	

**Table 5:** Embryonal development of sibling oocyte analysis for non-male factor patients.

Parameter	ICSI (n = 756)	IVF (n = 518)	*p*-Value
Number of oocytes	5.70 ± 3.35	5.60 ± 3.39	0.21
Decision regarding embryos			
Discarded	355 (47%)	280 (54%)	0.014
Freeze	259 (34.2%)	146 (28.2%)	0.02
Transfer	142 (18.8%)	92 (17.8%)	0.64
KIDScore	3.68 ± 1.51	3.35 ± 1.52	<0.001

**Table 6:** Treatment characteristics in the subgroup of sibling oocytes, according to the cause of infertility.

Variable *	PCOS n = 121		Endometriosis, n = 83		Mild Male Factor, n = 112		Unexplained, n = 544		Tubal Factor, n = 178	
	ICSI n = 70	IVF n = 51	ICSI n = 48	IVF n = 35	ICSI n = 80	IVF n = 32	ICSI n = 321	IVF n = 229	ICSI n = 96	IVF n = 82
Pregnancy	30 (42.9%)	18 (35.3%)	3 (6.3%)	3 (8.6%)	13 (16.3%)	5 (15.6%)	100 (31%)	66 (29%)	15 (15.6%)	12 (14.6%)
Discard	28 (40%)	32 (63%)	25 (52%)	24 (68%)	42 (52.5%)	15 (47%)	155 (48%)	124 (54%)	42 (44%)	35 (43%)
Freeze	32 (46%)	12 (23%)	17 (35%)	9 (26%)	30 (37.5%)	10 (31%)	92 (29%)	65 (28%)	36 (38%)	25 (30%)
Transfer	10 (14%)	7 (14%)	6 (13%)	2 (6%)	8 (10%)	7 (22%)	74 (23%)	40 (18%)	18 (18%)	22 (27%)
Endometrial thickness, mm	3.65 ± 1.5	3.62 ± 1.75	5.36 ± 3.2	5.69 ± 3.1	4.01 ± 1.4	3.87 ± 1.9	3.59 ± 2.3	3.76 ± 2.4	4.46 ± 2.6	4.51 ± 2.7
KIDScore	* 4.02 ± 1.38 (*p* < 0.001)	* 3.37 ± 1.54	3.65 ± 1.5	3.14 ± 1.4	3.66 ± 1.4	3.90 ± 1.2	3.51 ± 1.6	3.3 ± 1.5	3.84 ± 1.5	3.76 ± 1.5
Oocytes aspirated	12.3 ± 4.9	12.1 ± 5.3	19.1 ± 15.7	17.1 ± 15.6	17.8 ± 10.7	14.9 ± 8.3	11.6 ± 6.4	10.8 ± 5.9	10.7 ± 3.7	10.2 ± 3.6

Data are presented as mean ± SD or n (%). * Reasons for infertility.

## Data Availability

The data presented in this study are available on request from the corresponding author. The data is not publicly available due to privacy.

## Supplementary Materials

The following supporting information can be downloaded at: https://www.mdpi.com/article/10.3390/jcm14010244/s1, Table S1. Demographic parameters and treatment results of the women included in the sibling oocyte analysis. Table S2. Sperm Parameters for the IVF and ICSI groups.
