# Supplementary material for: Non-Male Factor Only—ICSI Can Overcome Oocyte Factor in PCOS Patients
Source: J Clin Med. 2025 Jan 3;14(1):244. doi: 10.3390/jcm14010244 (PMC11721918; doi:10.3390/jcm14010244)
Supplement: Supplementary file 1 [file jcm-14-00244-s001.zip › jcm-3310985-supplementary.pdf]

Supplementary Materials

Table S1. Demographic parameters and treatment results of the women included in the sibling oocyte analysis.

Parameter	Sibling oocyte subgroup
Age (years)	35.9±6.1 [20.88-44.9]
BMI ≥18 (kg/m²)	26.4±6.5
FSH (IU/L)	7.9±3.3
LH (IU/L)	6.07±3.8
E2 (pg/ml)	53.1 [35.5-119.6]
P (ng/ml)	0.26 [0.17-0.40]
**Infertility cause**	
PCOS	17 (8.2%)
Endometriosis	12 (5.7%)
Mild Male factor	15 (7.2%)
Unexplained	96 (45.9%)
Tubal factor	32 (15.3%)
Combined	37 (17.7%)
**Treatment Protocol**	
Antagonist	170 (81.7%)
Long agonist	14 (6.7%)
Short agonist	22 (10.6%)
Other	2 (1.0%)
Oocytes collected per woman	9.8±5.9 [2-49]
Pregnancy	54/210 (25.7%)

Table S2. Sperm Parameters for the IVF and ICSI groups.

Parameter	Both IVF groups (n=211)	ICSI group (n=1377)	Total (n=1588)	P value
Sperm volume, ml	2.0 [1-3]	1.8 [0.5-3]	1.80 [0.5-3]	0.067
Sperm concentration, IL/ml	45 [24-70]	20 [1.5-58]	25.00 [2.3-60]	**<0.001**
Sperm motility, %	50 [38-66]	42 [19.3-62.5]	43.00 [20-63]	**<0.001**
Sperm morphology, %	0 [0-0]	0 [0-0]	0 [0-0]	**0.011**

Median (IQR = interquartile range).
